# Improvement of the Digestibility of Sulfated Hyaluronans by Bovine Testicular Hyaluronidase: A UV Spectroscopic and Mass Spectrometric Study

**DOI:** 10.1155/2014/986594

**Published:** 2014-05-27

**Authors:** Katharina Lemmnitzer, Jürgen Schiller, Jana Becher, Stephanie Möller, Matthias Schnabelrauch

**Affiliations:** ^1^Institute of Medical Physics and Biophysics, Medical Faculty, University of Leipzig, Härtelstraße 16-18, 04107 Leipzig, Germany; ^2^INNOVENT e. V., Biomaterials Department, Pruessingstraße 27B, 07745 Jena, Germany

## Abstract

Glycosaminoglycans (GAGs) such as hyaluronan (HA) and chondroitin sulfate (CS) are important, natural polysaccharides which occur in biological (connective) tissues and have various biotechnological and medical applications. Additionally, there is increasing evidence that chemically (over)sulfated GAGs possess promising properties and are useful as implant coatings. Unfortunately, a detailed characterization of these GAGs is challenging: although mass spectrometry (MS) is one of the most powerful tools to elucidate the structures of (poly)saccharides, MS is not applicable to high mass polysaccharides, but characteristic oligosaccharides are needed. These oligosaccharides are normally generated by enzymatic digestion. However, chemically modified (particularly sulfated) GAGs are extremely refractive to enzymatic digestion. This study focuses on the investigation of the digestibility of GAGs with different degrees of sulfation by bovine testicular hyaluronidase (BTH). It will be shown by using an adapted spectrophotometric assay that all investigated GAGs can be basically digested if the reaction conditions are carefully adjusted. However, the oligosaccharide yield correlates reciprocally with the number of sulfate residues per polymer repeating unit. Finally, matrix-laser desorption and ionization (MALDI) MS will be used to study the released oligosaccharides and their sulfation patterns.

## 1. Introduction

Polysaccharides of the glycosaminoglycan (GAG) type such as hyaluronan (HA) or chondroitin sulfate (CS) are important constituents of the extracellular matrix (ECM) of connective tissues which is omnipresent in all vertebrates [1]. Since many inflammatory diseases such as rheumatoid arthritis are accompanied by degradation of the ECM, GAGs experience nowadays considerable medical and diagnostic interest [2]. Advances in “tissue engineering,” a hot topic of current biomedical research, have also significantly increased the scientific interest in GAGs [3].

Motivated by the discovery of the “sulfation code” [4], the interest in chemically (over)sulfated GAGs has also significantly increased because these compounds were shown to possess many beneficial properties and improve, for instance, the incorporation of metal implants into the corresponding tissue such as bone [5]. However, there are also negative aspects: chemically sulfated GAGs may be toxic, as evidenced by the lethal contamination of heparin with a chemically oversulfated CS [6], so caution is advised prior to the* in vivo* use of any chemically sulfated GAGs, including sulfated HA.

Although the overall degree of sulfation can be easily determined by elementary analysis (i.e., by the determination of the sulfur content of a GAG sample), a more detailed structural analysis of the sulfation pattern is a challenging task. Although ^13^C nuclear magnetic resonance (NMR) spectroscopy is one of the most powerful methods of structural GAG analysis, this method may easily fail for two reasons. First, the line widths of the ^13^C NMR resonances of GAG polysaccharides are significant and, thus, prevent the detection of small chemical shift differences [1]. Second, the introduction of sulfate residues reduces the achievable frequency dispersion of the carbon resonances of the dedicated GAG and, thus, causes considerable assignment problems [7].

Even though mass spectrometry (MS) is an additional powerful method of structure elucidation (particularly when combined with MS/MS and/or chromatographic separation) [5], the application of MS to polysaccharides is difficult and this particularly applies if polymers with charged functional groups such as sulfate (i.e., strong electrolytes) are of interest: while native dextran (with a molecular weight of about 67 kDa but without any charged groups) [8] is detectable by MS, the strongly sulfated heparin was not detectable in a mass higher than about 4 kDa [5].

Therefore, the most common way of mass spectrometric GAG analysis is based on previous enzymatic digestion of the polysaccharides: using enzymes such as testicular or bacterial hyaluronidases or bacterial chondroitinases (normally of the ABC type which is capable of cleaving CS A, B, and C), the native GAGs can be efficiently converted into characteristic oligosaccharides [1]. While chondroitinase ABC generates nearly exclusively a single Δ^4,5^ unsaturated disaccharide [9], the mechanisms of the hyaluronidase digest are more complex leading to a broad mixture of different oligosaccharides [10], whereby the hexasaccharide is normally the most abundant product [11]. The efficient digestibility of the naturally occurring GAGs is the most important reason why the corresponding oligosaccharides (particularly that of HA) are commercially available at moderate prices. Higher sulfated GAG oligosaccharides (with the exception of heparin) are, however, commercially less readily available. This is very unfortunate because such defined GAG oligosaccharides would be needed for several reasons: on the one hand, such compounds are required for binding studies with other molecules, particularly proteins, to reduce the complexity (in comparison to the native GAGs) of the system and to enable molecular modeling [12]. On the other hand, it has been recently shown that GAGs exhibit different properties depending on their molecular weights: while high mass HA has anti-inflammatory properties, HA oligosaccharides are known to exhibit proinflammatory properties, maybe due to the enhanced content of reactive end groups [13].

There are two reasons why sulfated oligosaccharides are commercially scarcely available. First, (over)sulfated GAGs (that are available from some animal tissues and/or can be synthesized by chemical sulfation of the native GAG polysaccharides) are less efficiently digested by enzymes such as chondroitinase or hyaluronidase [14]. Second, the chromatographic separation of mixtures of variously (over)sulfated GAGs is very challenging and cannot (to these authors' best knowledge) be routinely performed in larger quantities, even if the separation of native HA or CS oligosaccharides by liquid chromatography or capillary electrophoresis is not a major problem at all [15].

We will focus here particularly on the first problem: by using an adapted spectrophotometric assay as well as matrix-assisted laser desorption and ionization time-of-flight (MALDI-TOF) MS, we will show that enzymatic digestion of all (over)sulfated GAGs can be basically achieved if the reaction conditions are carefully adjusted. Nevertheless, the yield of digestion products decreases if the degree of sulfation of the polysaccharide educt increases.

## 2. Materials and Methods

### 2.1. Chemicals

Hyaluronan (HA, from* Streptococcus*, MW ≈ 1.1 × 10^6^ g mol^−1^) was obtained from Aqua Biochem Dessau, Germany; sulfur trioxide/dimethylformamide complex (SO_3_-DMF, purum, ≥ 97%, active SO_3_ ≥ 48%) and sulfur trioxide/pyridine complex (SO_3_-pyridine, pract.; ≥ 45% SO_3_) were from Fluka Chemie, Buchs, Switzerland. All other chemicals, solvents, and hyaluronidases from* bovine testes* (BTH; Type I-S, lyophilized powder, 400–1000 units/mg solid) were obtained in highest commercially available purity from Sigma-Aldrich (Deisenhofen, Germany) and used without further purification.

### 2.2. Synthesis and Analytical Characterization of Hyaluronan Sulfates

The hyaluronan sulfates (sHA1–sHA3) were synthesized as previously described by Hintze et al. [16]. In brief, in a first step the sodium salt of HA was transformed into its tetrabutylammonium salt (TBA-HA) using a Dowex WX8 ion exchanger. The following sulfation reactions of the TBA-HA were performed under argon in DMF at room temperature. For the syntheses of sHA1 (average number of sulfate groups per disaccharide repeating unit (ds_s_) = 1.2) and sHA2 (ds_s_ = 1.8) an SO_3_-pyridine complex (sHA1: molar polymer/SO_3_ ratio 1 : 6; sHA2: molar polymer/SO_3_ ratio 1 : 8) was used as sulfation agent. sHA3 (ds_s_ = 3.0) was sulfated using an SO_3_-DMF complex (molar polymer/SO_3_ ratio 1 : 20). The sulfated products were isolated from the reaction mixture by precipitation into acetone and neutralized with ethanolic NaOH solution. The formed sodium salts of the sHAs were washed with acetone and purified by dialysis against distilled water followed by lyophilization and drying of the resulting polymers under vacuum.

The degree of sulfation was determined by estimation of the sulfur content using an automatic elemental analyzer (Euro EA3000 CHNS, EuroVector, Redavalle, Italy). Molecular weight determination was performed by gel permeation chromatography (GPC) with a double detection system consisting of a Postnova Analytics PN 3000 (15°) laser-light scattering (LLS) detector and a Jasco RID-1531 refraction index (RI) detector. Absolute values of number-average (Mn) and weight-average (Mw) molecular weights were determined using the laser-light scattering (LLS) detection system. The calculation of the polydispersity (PD = Mw/Mn) was performed on the basis of Mn and Mw values obtained from RI detection. GPC operating parameters can be found in the work by Hintze et al. [16]. The most relevant analytical data are also summarized in Table 1.

### 2.3. Enzymatic Digestion of Differently Sulfated Hyaluronan Derivatives

Aqueous solutions of (a) native, that is, nonsulfated hyaluronan, and (b) three chemically sulfated hyaluronan derivatives with degrees of sulfation of 1.2, 1.8, and 3.0 were digested with hyaluronidase from* bovine testis* (BTH) using a final concentration of 0.4 mg/mL. The HA substrates (0.5 mg/mL, 0.5 mL) were dissolved in citrate-phosphate-buffer, consisting of 0.1 M citric acid adjusted with 0.2 M aqueous Na_2_HPO_4_ to the desired pH value of pH 5.7, and digested under continuous shaking for 20 hours at 37°C. For further optimization of the digestion conditions the sodium chloride concentration was varied between 0 and 0.5 M and the pH value between 3 and 9.

### 2.4. Determination of the Reactive End Groups by Photometry

The number of reducing (N-acetylglucosamine) end groups was used as a measure of the extent of the enzymatic digestion: when a GAG is digested, the absolute amount of the GAG remains indeed constant. However, the number of molecules increases. Each of the resulting oligosaccharides possesses a reducing end group and the number of the end groups can be conveniently determined. For the performing of the Reissig method [17, 18] two solutions were prepared. Solution (I) contained 4.94 g H_3_BO_3_ and 1.98 g KOH in 100 mL distilled water. Solution (II) comprised 5 g p-dimethylaminobenzaldehyde (DMAB) dissolved in 6.25 mL HCl (12 M). This solution was made up to 50 mL with glacial acetic acid in a graduated cylinder and once again diluted 1 : 10 (v/v) with glacial acetic acid immediately prior to use. The relative amounts of the digestion products indicated by the Reissig signal (absorbance at 585 nm) were monitored by a colorimetric assay according to Muckenschnabel et al. [18] modified by Asteriou et al. [19] and further modified in the following way: 100 µL of the digestion mixture (or the corresponding polysaccharide solution in the absence of the enzyme) was mixed with 200 µL of solution (I) to obtain a pH value of about 9 which is required for the Morgan-Elson reaction (described in detail in [18]). After heating at 95°C for 3 minutes the samples were cooled to 4°C. Afterwards, 1.5 mL solution (II) was added, thoroughly mixed, and placed in a shaker at 37°C for 15 min. The absorbance at 585 nm was subsequently determined on a Hitachi U-2000 spectrophotometer in 1.5 mL semimacro PMMA (poly-methyl-methacrylate) cuvettes against water. The absorbance at 585 nm was also determined for the same sample but without previous digestion and subtracted from the value determined in the presence of the enzyme. This difference is referred to as the Reissig signal and is directly proportional to the number of reducing N-acetyl-d-glucosamine end groups. The absorbance at 600 nm was determined as well to exclude potential influences of turbidity changes due to the digestion of long polysaccharide chains which may aggregate with the enzyme [20].

### 2.5. MALDI-TOF Mass Spectrometry

All MALDI-TOF mass spectra were acquired on an Autoflex mass spectrometer (Bruker Daltonics, Bremen, Germany) in the linear mode under delayed extraction conditions as previously described [10, 21]. Although mass spectra recorded in the linear mode have only limited resolution and a reduced mass accuracy [21], the higher sensitivity and the reduced generation of fragmentation products are clear advantages of this approach. The system utilizes a pulsed nitrogen laser, emitting at 337 nm. The extraction voltage was 20 kV and gated matrix suppression was applied to prevent the saturation of the detector by matrix ions [22].

200 single laser shots were averaged for each mass spectrum. The laser fluence was kept about five percent above threshold to obtain optimum signal to noise ratios.

Saturated 9-aminoacridine (9-AA) [23, 24] in methanol was used as matrix for negative ion detection. Spectra were analyzed with the program FlexAnalysis.

## 3. Results and Discussion

The GAGs of particular interest for this communication are (a) native (nonsulfated) hyaluronan and (b) chemically sulfated HA derivatives. However, the polymer repeating units remain unchanged upon derivatization: the disaccharide unit of HA is composed of a d-glucuronic acid, which is 1,3-glycosidically linked to N-acetyl-d-glucosamine. These disaccharides are 1,4-glycosidically linked with each other forming a linear (nonbranched) polysaccharide with a molecular weight (depending on the biological source the HA sample is isolated from) reaching up to several hundred kilodalton (kDa) [25]. The chemical sulfation is known to introduce additional sulfate residues preferentially at the C-6 of the N-acetyl-d-glucosamine unit and (under more vigorous reaction conditions) at the C-4 of the latter unit and the C-2 and C-3 of the d-glucuronic acid residue. The number of sulfate residues per disaccharide unit depends on the reaction conditions and may be up to four. This is termed ds_s_ = 4 and “ds_s_” denotes the average number of sulfate groups per disaccharide repeating unit. We will use here HA derivatives with a reduced extent of sulfation.

**Figure 1:**
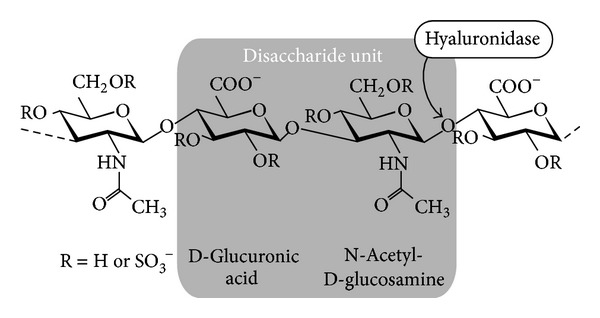
Chemical structure of native (R = H) and chemically sulfated HA (R = SO_3_^−^). The cleavage site of hyaluronidase (exclusively the 1-4 glycosidic linkages) is also illustrated in the figure. Note that the sulfate residue represents a strong electrolyte; that is, it always exists in the deprotonated form.

Figure 1 shows a schematic survey of the structures of the GAGs relevant to this study. The analytical data of the synthesized hyaluronan sulfates are summarized in Table 1.

Hyaluronidase from* bovine testis* (BTH) is known to act as a hydrolase and cleaves the 1,4-glycosidic linkages in the hyaluronan polysaccharide nearly randomly [26]. We will focus on this enzyme because BTH is commercially available at a very moderate price. Additionally, BTH also hydrolyses chondroitin, chondroitin-4-sulfate and chondroitin-6-sulfate, as well as dermatan (sulfate) and its application is, thus, not limited to the degradation of HA [27].

The Reissig signal [17, 18] as a measure of the number of free N-acetyl-d-glucosamine end groups was determined before and subsequent to enzymatic digestion of the differently sulfated HA samples. In order to make the data more obvious, the difference (Δ-absorbance) between both values at 585 nm will be shown as a relative measure of the extent of the related enzymatic activity

**Figure 2:**
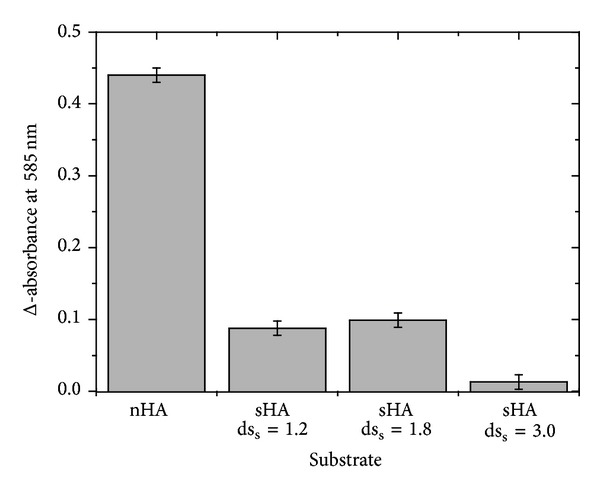
Dependence of the Reissig absorption (difference of the absorption at 585 nm without and subsequent to digestion with BTH) on the used hyaluronan samples. All samples were digested with BTH for 20 hours at 37°C at pH 5.7. No salts beside the buffer components were added. For details see text.

Figure 2 clearly indicates that the change in the absorbance at 585 nm depends significantly on the substrate and, thus, the degree of sulfation of the used polysaccharide: the digestion of the native HA results in the highest increase of the absorbance; that is, a maximum of oligosaccharides is obtained under these conditions. The sulfated HA derivatives with ds_s_ = 1.2 (sHA1) and 1.8 (sHA2) are digested (within the experimental error range) to the same extents; however, only about 20% of the effect that is detectable in the case of the native HA can be achieved. That is, the extent of digestion is significantly reduced when sulfated HA samples are used as substrates [5, 14]. Considering the accuracy of the applied method, the very low absorbance detected in the case of the highly sulfated derivative (sHA3, ds_s_ = 3) suggests that no significant degradation has taken place. Therefore, BTH is only capable of digesting the moderately sulfated HAs albeit only much smaller yields of oligosaccharides can be obtained when compared to the native HA.

The exhaustive enzymatic digestion of (sulfated) HAs leads to tetra-, hexa-, and octasaccharides as the most abundant products [10]. Of course, these products can be easily differentiated depending on the number of sulfate residues per oligosaccharide, that is, by their molecular weights.  Table 2 shows the (theoretical) atomic masses of HA oligosaccharides generated from polymeric HA by digestion with BTH. In addition to nonsulfated HA, sulfated HA derivatives with varying numbers of sulfate groups are also considered in Table 2.

**Figure 3:**
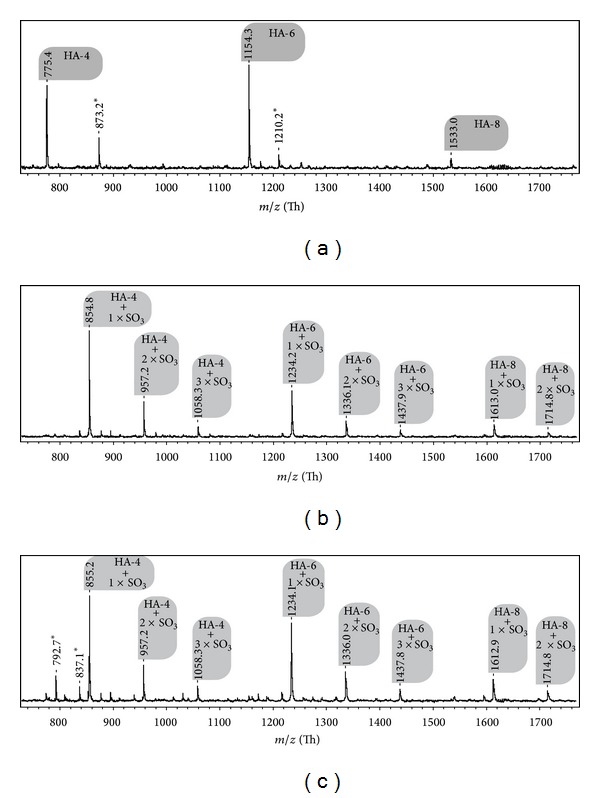
Negative ion MALDI-TOF mass spectra of differently sulfated HA samples subsequent to digestion with hyaluronidase but without any further purification of the reaction mixture. Trace (a) corresponds to native HA, while (b) and (c) represent HA with ds_s_ = 1.2 and 1.8, respectively. All spectra were recorded in the linear mode of the MS device and in the presence of 9-aminoacridine as matrix with a sample to matrix ratio of 2 to 1 (v/v). This corresponds to a 25-fold weight excess of the matrix. Peaks stemming from impurities are marked by asterisks. Please note that the achievable mass accuracy is only of the order of about 200 ppm. This is a rather typical value if the linear (but not the reflector modus) is used and the reason why only one decimal is given.

Soft ionization mass spectrometric techniques such as MALDI or ESI are convenient methods to investigate the molecular weights of oligosaccharides [28]. We will focus here on MALDI MS because this method is influenced to a lesser extent by sample impurities, particularly the salt content. In the negative ion MALDI-TOF mass spectra shown in Figure 3 all detected peaks can be easily assigned to the corresponding oligosaccharides (cf.  Table 2). As expected, native HA is primarily digested into the tetra-, hexa-, and octasaccharides which can be easily differentiated (cf. trace (a) of Figure 3). The mass difference of one unit in comparison to the data given in Table 2 is caused by the loss of one proton which is necessary to make the oligosaccharide detectable as a negative ion.

In the case of the sulfated HA with ds_s_ = 1.2 and 1.8, however, only sulfated oligosaccharides are detectable, whereby the tetra-, hexa-, and octasaccharides with one to a maximum of three sulfate residues (cf. Figure 3, traces (b) and (c)) can be identified. The observed mass differences of 22 or 44 units (cf.  Table 2) are caused by H^+^/Na^+^ exchange. These differences do exclusively occur if the spectra of sulfated HA oligosaccharides are considered. Although a detailed investigation of this aspect is beyond the scope of this paper, this difference might be caused by either (a) the strongly different pK values of sulfate in comparison to carboxylate [1] or (b) an enhanced salt content within the chemically modified HA samples.

Unfortunately, the digestion of the HA sample with the most significant extent of sulfation (sHA3, ds_s_ = 3.0) did not result in an oligosaccharide concentration which could be detected by MALDI MS. However, it should be explicitly noted that oligosaccharide concentrations below the detection limits are only one potential explanation: in addition to this aspect, charged saccharides are always more difficult to detect by means of MS than neutral saccharides. This particularly applies to carbohydrates with strong electrolytes such as sulfate [29].

**Figure 4:**
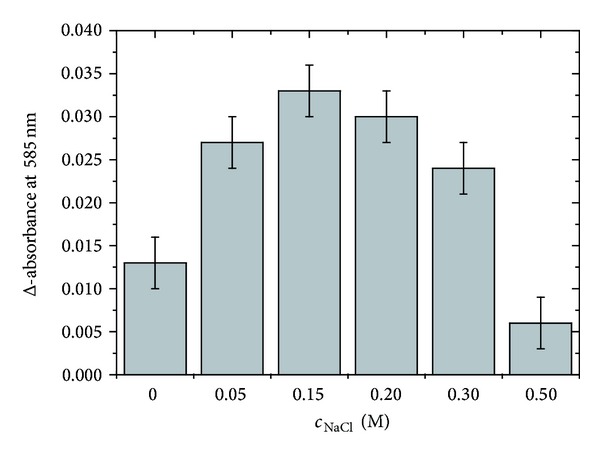
Dependence of the Reissig absorption (as a measure of the reducing end groups, i.e., the extent of the enzymatic digestion) on the NaCl concentration. Since the HA sample with the highest sulfate content (ds_s_ = 3) was most refractive to the hyaluronidase digestion, only this sample was investigated. All samples were digested with BTH for 20 hours at 37°C at pH 5.7 prior to measurements.

In order to improve the extent of the enzymatic degradation of the HA with the most significant extent of sulfation, the digestion conditions were further optimized: the dependence of the Reissig signal (subsequent to BTH digestion) on the used NaCl concentration is shown in Figure 4. It is obvious that the maximum of digestion products is obtained when 0.15 M NaCl is used, that is, when the digestion is performed at physiological salt concentrations. This improved digestibility is presumably caused by the (partial) shielding of the negative charges of the HA polysaccharide by the sodium ions.

**Figure 5:**
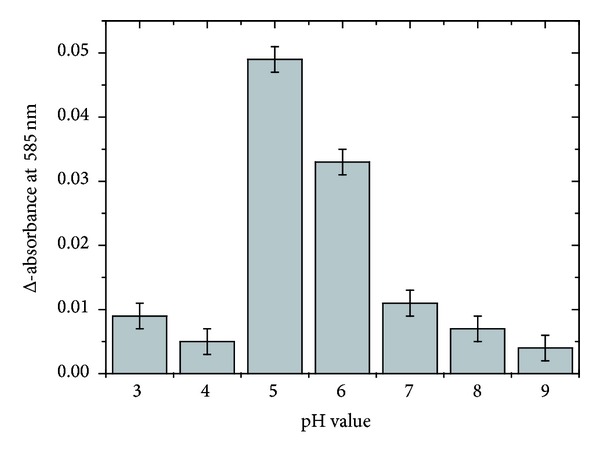
Dependence of the Reissig absorption on the pH value of the digestion mixture. Since the HA sample with the highest sulfate content (sHA3) was most refractive to the hyaluronidase digestion, only this sample was investigated. All samples were digested with BTH for 20 hours at 37°C and physiological NaCl concentration (0.15 M).

In addition to the salt concentration, the pH of the reaction mixture was also systematically varied to evaluate the optimum conditions of the enzymatic digestion, whereby the NaCl concentration was kept constant at 0.15 M. The achieved data (given in Figure 5) unequivocally indicate that a pH value of about 5 represents the optimum reaction conditions to obtain a maximum of enzymatic digestion products, while reduced product yields are obtained at slightly lower or higher pH values.

**Figure 6:**
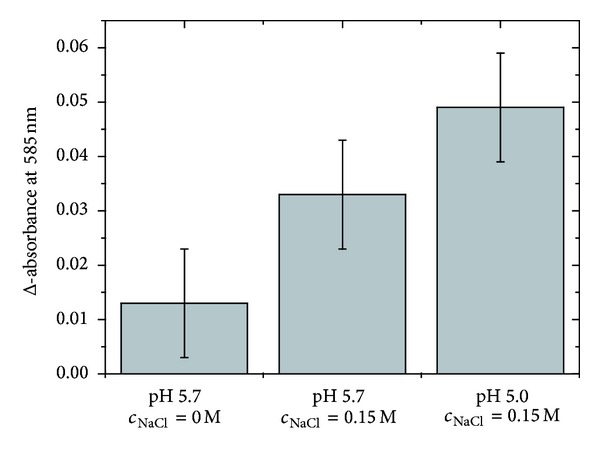
Determination of the Reissig absorption under the optimized reaction conditions discussed above. HA with ds = 3 was exclusively used. The applied conditions are indicated directly in the figure. Error bars represent the systematic error of the performed measurements.

All data related to the digestion of the HA sample with the highest sulfate content (sHA3) are finally summarized in Figure 6.

The comparison of the Reissig signal intensities after the three different digestions of the highly sulfated HA derivative (Figure 6) provides clear evidence that the yield of the oligosaccharides can be fourfold increased in comparison to the originally used conditions (no NaCl addition and a pH of 5.7) if a sodium chloride concentration of 0.15 M and a pH value of 5 are applied to digest the HA samples.

**Figure 7:**
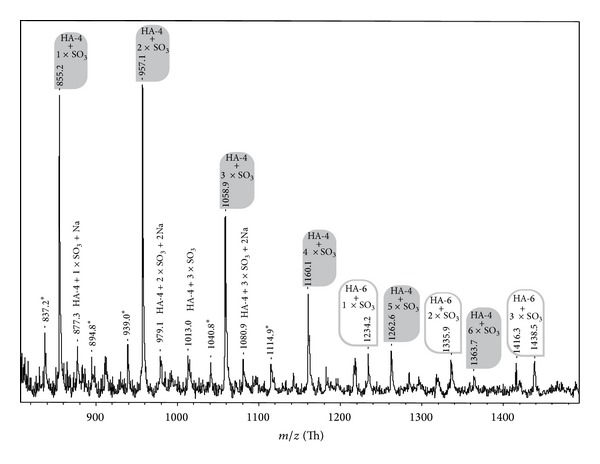
Negative ion MALDI-TOF mass spectrum of the sulfated HA with ds_s_ = 3.0 subsequent to digestion with BTH at optimized conditions but without further purification of the reaction mixture. All spectra were recorded in the linear mode of the MS device and in the presence of 9-aminoacridine as matrix using a sample to matrix ratio of 2 to 1 (v/v). Sulfated tetrasaccharides are marked by grey and hexasaccharides by white bars, respectively. Obvious impurities are marked by asterisks.

Although this might be considered as a minor progress only, this yield difference is sufficiently pronounced to enable the MALDI MS characterization of the related oligosaccharides: the negative ion MALDI-TOF mass spectrum of the HA polysaccharide with the most pronounced sulfate content (ds_s_ = 3.0) subsequent to BTH digestion at the optimized conditions is shown in Figure 7. In addition to the products already illustrated in the context of Figure 3, additional peaks at* m/z* 1262.6 and 1363.7 are obvious. Although these peaks possess minor intensities and there is slight interference with the corresponding hexasaccharide, these signals can be easily assigned to the HA tetrasaccharide with 5 and 6 sulfate residues, respectively.

The reader should note that the peak intensities do not necessarily correlate with the concentrations of the individual sulfated oligosaccharides [22]: in addition to the molecular weight of a compound, the number of the sulfate residues does also (negatively) affect the peak intensities. An additional problem is that there is a pronounced tendency of the sulfate residues to be lost in the gas phase [30] and the probability of sulfate loss increases with the number of sulfate residues. This problem can be minimized but never completely suppressed. Since electrospray ionization (ESI) MS is known to represent an even more gentle ionization technique than MALDI MS [31], we have also attempted to use ion trap (IT) ESI MS for the characterization of the individual samples. However, these attempts were so far not successful. We believe that the salt content (even subsequent to dilution) of the samples suppresses the successful ion generation within the ESI source because ESI MS is much more sensitive to contaminations when compared to MALDI MS [5].

Although we are aware of these obvious problems, the present study should be considered as a pioneering work: to our best knowledge it was so far not possible at all to convert highly sulfated HA samples into the corresponding oligosaccharides. Even if further improvements are obviously necessary, this study has provided sufficient evidence that strongly sulfated hyaluronans are basically digestible by bovine testicular hyaluronidase.

## 4. Conclusions

We have shown that chemically sulfated hyaluronan derivatives can be digested by bovine testicular hyaluronidase if the conditions of the enzymatic digestion are carefully adjusted, although the oligosaccharide yield is still strongly dependent on the number of sulfate residues per polymer repeating unit.

The molecular weights of the generated oligosaccharides were additionally verified by using MALDI-TOF mass spectrometry. All of the described experiments were exclusively performed on an analytical scale. However, our next step will be the extension of this study to obtain the oligosaccharides in at least mg amounts. If successful, this would be an important progress because the availability of GAG oligosaccharides with defined sulfation patterns is so far extremely limited.

## Figures and Tables

**Table 1:** Selected characteristics of the synthesized HA derivatives.

Sample	sHA1	sHA2	sHA3
ds_s_	1.2	1.8	3.0
*M*_*n*_ (g × mol^−1^)	15 910 (39 540)	16 673 (43 993)	23 725 (29 275)
*M*_*w*_ (g × mol^−1^)	26 790 (87 410)	32 370 (86 150)	29 525 (48 340)
PD	2.2	2.0	1.7

ds_s_, number-average (*M*_*n*_), and weight-average (*M*_*w*_) molecular weights as determined by laser-light scattering (LLS) and refractive index (RI) (in parentheses) detection. Molecular weight distributions (polydispersity index (PD)) were determined based on the values calculated from RI detection.

**Table 2:** Mass list of the most abundant digestion products of HA after exhaustive digestion with bovine testicular hyaluronidase.

Hyaluronan oligosaccharide	Monoisotopic molecular masses			
	Number of sulfate residues			
	0	1	2	3
Tetrasaccharide (HA-4)	776.23	856.19	936.15	1016.10
Hexasaccharide (HA-6)	1155.34	1235.30	1315.26	1395.22
Octasaccharide (HA-8)	1534.46	1614.41	1694.37	1774.33

The table includes the masses of sulfated derivatives with up to three sulfate residues per oligosaccharide. Please note that all data were calculated by using the monoisotopic masses. Since the data will be used to explain the mass spectra (vide infra), charge compensation by protonation (not by alkali metal ions) is exclusively assumed to occur.
